# Prediction of protein–protein interaction using graph neural networks

**DOI:** 10.1038/s41598-022-12201-9

**Published:** 2022-05-19

**Authors:** Kanchan Jha, Sriparna Saha, Hiteshi Singh

**Affiliations:** 1 Department of Computer Science and Engineering, Indian Institute of Technology Patna, Patna, Bihar 801103 India; 2 Department of Electrical Engineering, Indian Institute of Technology Jodhpur, Jodhpur, Rajasthan 342030 India

**Keywords:** Computational biology and bioinformatics, Computational models

## Abstract

Proteins are the essential biological macromolecules required to perform nearly all biological processes, and cellular functions. Proteins rarely carry out their tasks in isolation but interact with other proteins (known as protein–protein interaction) present in their surroundings to complete biological activities. The knowledge of protein–protein interactions (PPIs) unravels the cellular behavior and its functionality. The computational methods automate the prediction of PPI and are less expensive than experimental methods in terms of resources and time. So far, most of the works on PPI have mainly focused on sequence information. Here, we use graph convolutional network (GCN) and graph attention network (GAT) to predict the interaction between proteins by utilizing protein’s structural information and sequence features. We build the graphs of proteins from their PDB files, which contain 3D coordinates of atoms. The protein graph represents the amino acid network, also known as residue contact network, where each node is a residue. Two nodes are connected if they have a pair of atoms (one from each node) within the threshold distance. To extract the node/residue features, we use the protein language model. The input to the language model is the protein sequence, and the output is the feature vector for each amino acid of the underlying sequence. We validate the predictive capability of the proposed graph-based approach on two PPI datasets: Human and *S. cerevisiae*. Obtained results demonstrate the effectiveness of the proposed approach as it outperforms the previous leading methods. The source code for training and data to train the model are available at https://github.com/JhaKanchan15/PPI_GNN.git.

## Introduction

Proteins are organic macromolecules made up of twenty standard amino acids. They are responsible for performing nearly all biological processes and cellular functions in organisms^1^. DNA transcription and replication, hormone regulation, metabolism, molecular cell signaling, and signal transduction are some examples of life activities that involve protein interactions^2,3^. Furthermore, the knowledge of PPI has proven to be helpful in new drug discovery as well as the prevention and diagnosis of diseases^4^. However, they rarely carry out their functions in isolation but interact with other proteins in their surroundings and accomplish their tasks. There are several high-throughput experimental methods such as Yeast two-hybrid screens (Y2H)^5^, Tandem affinity purification (TAP)^6^, and Mass spectrometric protein complex identification (MS-PCI)^7^ which are used to identify the interaction between proteins. These experimental methods contributed to the creation of PPI datasets for different species but at a slow speed. Moreover, as the experimental environment and device resolution influence the output, the PPI data collected by these methods have a high rate of false positives and false negatives^8,9^. When used in conjunction with experimental methods, high-throughput computational methods improve the accuracy and quality of PPI prediction^10^.

To date, there have been a plethora of works that employed traditional machine learning (ML) techniques to solve several problems in the computational biology domain, such as protein classification, structure prediction, protein interaction prediction, etc. Support vector machines (SVMs), Random forest (RF), Neural networks (NNs) are widely used ML algorithms to classify protein interactions. The inputs to these algorithms are hand-engineered features, which are mainly derived from underlying protein sequences using physicochemical properties, evolutionary information, and distribution patterns of amino acids. For example, Shen et al.^11^ have employed a conjoint triad feature extraction method, which divides 20 amino acids into seven groups based on their properties, and SVM with a kernel function as a learning algorithm. Guo et al.^12^ have used the autocovariance (AC) method to encode protein sequences and SVM as a classifier to predict PPI. AC method is intended to consider the neighboring effect by capturing the physicochemical properties of residues a certain distance apart in the sequence. Li et al.^13^ have developed a method incorporating a mixture of evolutionary features based on a Position-specific scoring matrix (PSSM) and physicochemical properties. The discriminative vector machine (DVM) is used as a classifier to predict protein interactions. Huang et al.^14^ have adopted the global encoding representation of proteins with a weighted sparse representation-based classifier to classify PPI. Li et al.^15^ have proposed a sequence-based method to predict self-interacting proteins. Firstly, the known protein sequences are converted into a Position-specific scoring matrix (PSSM), and then Low-Rank Approximation (LRA) is used to get feature vectors from PSSM. Finally, the extracted vectors are the inputs to the rotation forest classifier, which distinguishes self-interacting and non-self-interacting proteins. The gradient boosting decision tree algorithm has been introduced to predict PPI by Zhou et al.^16^. This algorithm is based on several protein descriptors such as frequency, composition, transformation, distribution, and autocovariance to encode protein sequences. Apart from sequence-based features, we have other sources of information to get input features for PPI models, including gene fusion^17^, protein structure^18^, function^19,20^, gene expression profile^21^, etc. Ding and Kihara^22^ reviewed and classified several computational methods based on input features mentioned above. Among all sources of proteins, sequence-derived features are the most commonly used to predict PPI.

Researchers have recently carried out considerable works on PPI using the latest deep learning techniques, which improve the model’s performance. These techniques allow them to use high-dimensional and complex input features. Sun et al.^23^ have used a stacked autoencoder to get compact and relevant representation of sequence-based input features. Du et al.^24^ have proposed a deep learning-based PPI model known as DeepPPI, which learns the high-level features from protein descriptors and outperforms the traditional ML algorithms. Hashemifar et al.^25^ have developed a model known as DPPI, which consists of three modules: siamese-like CNN, random projection, and prediction. The input to the DPPI is the sequence profiles of proteins in a pair, and the output is the binary value representing whether they will interact or not. Gonzalez-Lopez et al.^26^ have proposed a PPI model in which features are learned using NLP techniques such as embedding and recurrent neural networks from raw protein sequences. They have shown that the state-of-the-art results can be obtained using only raw data without relying on feature engineering to predict protein interactions. EnsDNN (Ensemble deep neural network) is a very complex PPI model proposed by Zhang et al.^27^. It uses autocovariance (AC), local descriptor (LD), and multi-scale continuous and discontinuous local descriptor (MCD) methods to get different feature representations of protein sequences. These feature vectors are then individually fed to the nine independent neural networks that differ in the number of layers and neurons, which produce a total of 27 neural networks. The output of each neural network is then fed to the multi-layer perceptron model having two hidden layers to predict the labels. A deep multi-modal framework, which utilizes structural and ontology-based features, is proposed by Jha et al.^28^ to predict the protein interactions and outperform the existing works.

Most of the above-mentioned deep-learning methods have only considered sequence-based features. The PPI models based on structural information of proteins are significantly less explored. Nowadays, the latest deep learning techniques, such as deep convolutional neural networks (CNN), have been used widely because of their potential to extract features seamlessly from structural data. For example, in our previous work^29^, we have used structural information with sequence-based features to predict the interactions between proteins. We have utilized a pre-trained ResNet50 model to extract structural features from the 2D volumetric representations of proteins. The obtained results suggest that techniques for image-related tasks can be extended to work for protein structures. But these approaches of analyzing molecular structure have certain issues such as high computing cost and interpretability.

Graph neural networks have made significant progress in recent years and have emerged as key tools in graph-based applications. For example, Huang et al.^30^ have predicted the associations between miRNA and drug resistance using graph convolution. The problem of predicting associations between them is formulated as a link prediction problem. Other applications of graph neural networks include prediction of chemical stability^31^, protein interface prediction^32^, protein solubility prediction^33^ and modeling the side effects of polypharmacy^34^. The prediction of interactions between proteins using the graph-based technique can be implemented in two ways: molecular structure-based and PPI network-based. Yang et al.^35^ have proposed the signed variational graph auto-encoder (S-VGAE) to predict the interactions between proteins by considering the PPI network as an undirected graph. This representation learning model can effectively use the graph structure and seamlessly assimilate protein sequence information as features. The current work explores the applicability of graph-based neural networks to predict protein interactions utilizing the graphical molecular representations of proteins. This representation of proteins allows for a natural spatial representation of molecular structures and is more interpretable than 2D volumetric representation.

In this paper, we propose a framework that combines graph-based techniques and language models (*SeqVec*^36^ and *ProtBert*^37^) to predict PPI. The molecular graph of a protein has nodes representing the amino acids of which proteins are made up of. The language model is used here to get features for each residue (node in graph) directly from protein sequences. The advantage of using the language model-based feature vectors is that it does not require domain knowledge to encode the sequences. The obtained results show its applicability in predicting protein interactions. We use graph-based methodologies, such as graph convolutional network (GCN)^38^ and graph attention network (GAT)^39^, to learn features from protein representations combining structural and sequence information. Finally, the feature vectors of proteins in pairs are concatenated and fed to the classifier having two hidden layers and an output layer. The following are the key contributions of this work:

1. We use the graphical representation of proteins with residues as nodes. We believe that it will improve the model’s performance as it covers spatial structural/low-level properties.
2. We use pre-trained language models (*SeqVec* and *ProtBert*) to get the feature vector for each residue/node in the constructed graph. We show that this feature vector is more beneficial than other features such as physicochemical properties of residues, one-hot encoding of amino acids, etc.
3. We propose two graph-based architectures: GCN-based and GAT-based to learn features from protein representation integrating spatial structure and sequence features. The obtained results propound the superiority of the proposed method over the existing works.

## Materials and methodology

The proposed methodology to predict PPI consists of three modules: protein graph construction, feature extraction, and classifier to predict interactions between them. This section discusses each module and the deep learning techniques used to implement them in detail. In addition, we describe the datasets used to evaluate the performance of our approach. All experiments were performed in accordance with relevant guidelines and regulations.

### Datasets

In this work, we have used the PPI datasets of two organisms: Human and *S. cerevisiae*. The Pan’s human dataset^40^ is available at http://www.csbio.sjtu.edu.cn/bioinf/LR_PPI/Data.htm. The positive pairs of this dataset are collected from the human protein reference database (HPRD, 2007 version). The elimination of duplicate and self-interacting pairs gives a total of 36,545 instances. For the *S. cerevisiae*, there are a total of 22,975 interacting protein pairs, downloaded from the Database of Interacting Proteins (DIP; version 20160731). After performing the preprocessing, such as removing protein pairs that have a protein with fewer than 50 amino acids, we have a total of 17,257 interacting pairs. The negative pairs for both organisms are generated by randomly pairing proteins with different subcellular localization. These generated negative instances should not be in the positive PPI dataset. The information regarding the subcellular localization of proteins is collected from the Swiss-Prot database. The Pan’s human negative PPI dataset has a total of 36,323 non-interacting samples consisting of pairs generated using the method discussed above and the negative instances from the Negatome database^41^. For *S. cerevisiae*, there are a total of 48,594 negative pairs. The CD-HIT^42^ tool with a sequence identity level of 40% as the cutoff has been used to remove protein pairs that are considered to be homologous (having too much sequence identity).

The 3D structures of all proteins utilized in this study are downloaded in PDB format from the RCSB Protein Data Bank (https://www.rcsb.org). It limits the number of samples in both PPI datasets as the structural information is not available for all proteins. The final statistics of these datasets are reported in Table 1.

**Table 1:** Characteristics of PPIs datasets.

Dataset	# Samples	# Positive samples	# Negative samples
Human	22,217	16,220	5997
*S. cerevisiae*	7274	2847	4427

### Graph representation of proteins

In this work, we have constructed the molecular graph of proteins, also known as amino-acids/residues contact network, using the PDB files. The PDB file is a text file containing structural information such as 3D atomic coordinates. Let *G*(*V*, *E*) be a graph representing the proteins, where each node ($v \in V$) is the residue and interaction between the residues is described by an edge ($e \in E$). Two residues are connected if they have any pair of atoms (one from each residue) having the Euclidean distance less than a threshold distance. The cutoff distance used in the literature is 6 angstroms (Å)^32^, and we are also using the same value for threshold distance.

#### Node features

Each node in the protein’s graph has some properties associated with it. These node features are mainly obtained from protein sequences and structures. In this work, the node features are extracted from protein sequences employing two pre-trained language models (LSTM-based^36^ and BERT-based^37^). In addition, we have also considered some other methods to get node features, such as one-hot encoding of 20 standard amino acids and physicochemical properties of residues. In the case of the one-hot encoding method, each node is represented as a vector of length 20. The seven physicochemical properties of amino acids provided by Meiler et al.^43^ are assumed to influence the interactions between proteins by creating hydrophobic forces or hydrogen bonds between them. So, these features are used as other feature vectors for each node in the graph. Table 2 reports the dimension of the node’s features obtained using the aforementioned techniques. Among these node features (based on the results obtained), the protein graph with node features extracted using the pre-trained LSTM-based language model (LM) outperforms the methods based on other feature vectors. The results are discussed in the subsection *Performance of GNN Variants using Different Node Features*.

**Figure 1:**
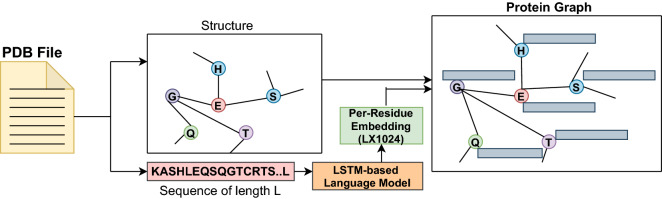
Graph representation of a protein with node features.

Figure 1 depicts all the steps of generating a protein graph from a PDB file. The first step is to get each protein’s PDB sequence and molecular graph structure using a python script. Then the residue-level features are merged with a molecular graph, creating a final protein graph.

**Table 2:** Size of node’s features.

S. no.	Method	Dimension
1	LSTM-based language model (*SeqVec*)	1024
2	BERT-based language model (*ProtBert*)	1024
3	One-hot encoding of amino acids	20
4	Physicochemical properties of amino acids	7

### Graph models

CNN-based models work effectively as feature extractors. But the limitation with these models is that they can only operate on regular Euclidean data like 2D grid images and 1D sequences. To work on non-Euclidean data, such as graph data, a graph neural network (GNN) has been developed, which can directly process the graph. Over the years, there has been a rapid development in GNN and has offered several variants^44^. This work explores the two popular GNN variants, GCN^38^ and GAT^39^, to extract features from proteins represented as graphs.

#### Graph convolutional network

The GCN is used to learn features from data represented as graphs. Let $P = (V, E)$ be a graph representation of a protein, where the number of vertices is the length of the PDB sequence (i.e., $\vert V \vert = L$). The connection between every pair of nodes is encoded in the adjacency matrix, $A \in R^{L, L}$. The matrix, $X \in R^{L, F}$, contains the residue-level features for all nodes of the given graph (*F*: dimension of node features). Each layer of GCN takes the adjacency matrix (*A*) and node embeddings from the previous layer ($H^{(l)} \in R^{L, F_l}$) as input and outputs the node-level embeddings for the next layer ($H^{(l+1)} \in R^{L, F_{l+1}}$).

1 $$\begin{aligned} H^{(l+1)} = GC(H^{(l)}, A) \end{aligned}$$

Here, $H^{(0)}$ = $X \in R^{L, F}$, $F_l$ and $F_{l+1}$ represent the dimensions of node-level embeddings for layers *l* and $l+1$, respectively. The more specific expression of Eq. (1), as defined by Kipf and Welling^38^ is:

2 $$\begin{aligned} H^{(l+1)} = ReLU(\hat{D}^{-0.5}\hat{A}\hat{D}^{-0.5}H^{(l)}W^{(l+1)}) \end{aligned}$$

Here, $\hat{A}$ is the adjacency matrix added with identity matrix $I_L \in R^{L,L}$ ($\hat{A} = A+I_L$). The addition of identity matrix to adjacency matrix enforces the self-loops in the graph, ensuring the inclusion of node’s own features in the sum during convolution operation. $\hat{D}$ is the diagonal node degree matrix calculated as: $\hat{D}_{ii} = \sum _{j=1}^{L}{\hat{A}_{ij}}$. It is used to normalize the adjacency matrix in a symmetric manner $(\hat{D}^{-0.5}\hat{A}\hat{D}^{-0.5})$ so that we get the normalized residue features after each convolutional layer. After the GCN layer, each residue feature vector is updated as a weighted sum of the features of neighboring nodes in the graph, including residue’s own feature (Eq. 2). $W^{(l+1)}$ is the trainable weight matrix.

#### Graph attention network

GAT^39^ is an attention-based architecture that operates on graph-structured data. The idea is to use a self-attention method to compute the hidden representation of each node in the graph by paying attention to its neighbors. In GAT, for input feature matrix $X \in R^{L, F}$, we will get the learned feature matrix $H \in R^{L, F'}$ as an output. Here, *L* is the number of nodes in the graph, and *F* and $F'$ are the input and output dimensions, respectively. The expression to calculate the learned feature of node *i* is:

3 $$\begin{aligned} H_i = \sigma \Bigg ( \sum _{j \in N_{i}}{\alpha _{ij}WX_{j}}\Bigg ) \end{aligned}$$

Here, $\sigma$ is the LeakyReLU activation function used to apply non-linearity to aggregated features of neighboring nodes, $N_i$. *W* is the weight matrix used to apply a linear transformation to the input feature matrix, *X*. The normalized attention coefficient between node *i* and *j*, represented as $\alpha _{ij}$, is calculated as:

4 $$\begin{aligned} \alpha _{ij} = \frac{e^{a(H_i, H_j)}}{\sum _{k \in N_{i}}{e^{a(H_i, H_k)}}} \end{aligned}$$

where $a \in R^{2F'}$ is a weight vector. Let $H \in R^{L, F'}$ be the learned feature matrix of protein graph *P* after one GNN layer. Here, $F'$, the dimension of a node’s feature, is fixed for all graphs’ nodes. But, *L*, which represents the number of nodes in a graph, may vary as each protein has different number of residues. To ensure the fixed-size representations of all proteins, we have added a global pooling layer after the GNN layer. The pooling layer is calculated by performing some operation (mean, max, sum) on the GNN layer’s output ($H \in R^{L, F'}$) across the number of nodes. In our case, we use the mean pooling to get a fixed size $(1, F')$ representation of all proteins, independent of the number of nodes *L* in a graph.

**Figure 2:**
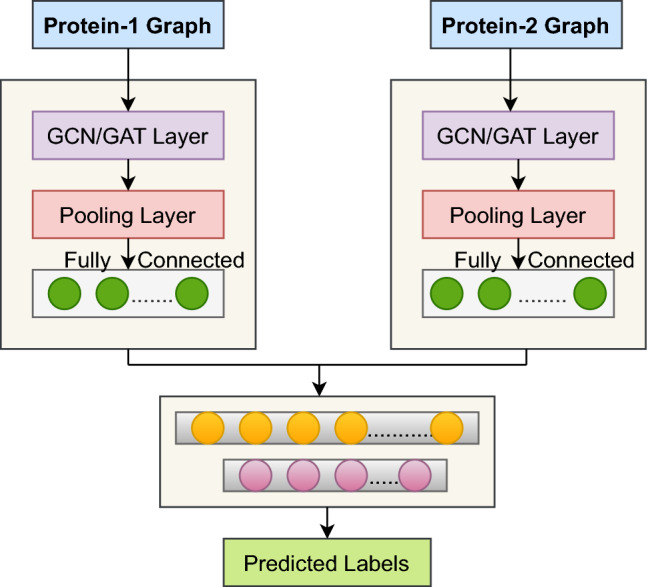
Illustration of the proposed approach.

After the pooling layer, we add a fully connected (FC) layer with a LeakyReLU activation function to get the final representation of a protein from its pooled representation. Similarly, we get the learned feature vector of another protein in a pair. The concatenated feature vectors of proteins in a pair are then fed to the classifier having two FC layers and an output layer. To avoid over-fitting, we add a dropout layer with a dropout rate of 0.2 after each FC layer. The output layer is the sigmoid layer, which determines whether the given pairs are interacting (≥ 0.5) or non-interacting $(<0.5)$. The LeakyReLU is used as an activation function to add non-linearity to the output of a fully connected layer. Figure 2 depicts the overall steps of the proposed methodology.

### Language models

**Figure 3:**
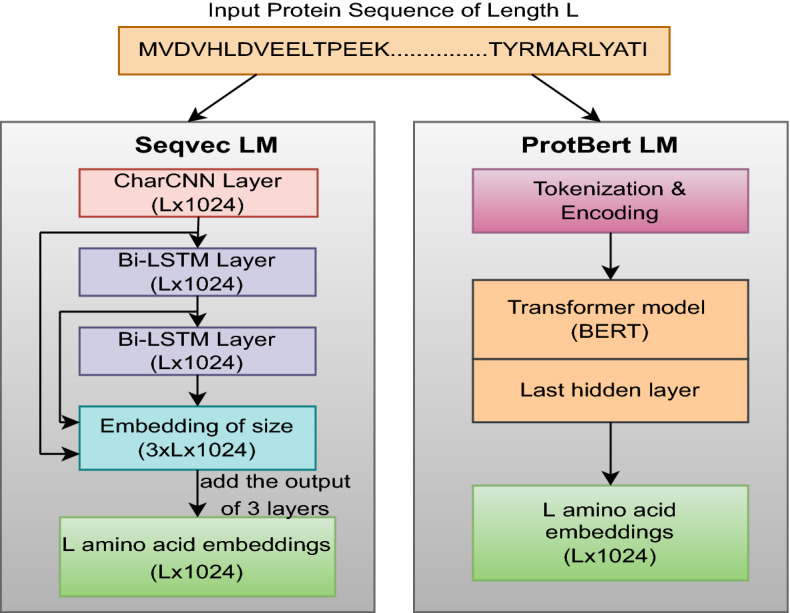
Illustration of the feature extraction from protein sequence using language models.

Proteins are the long chain of amino acids, where each amino acid (residue) can be considered as a word and each sequence as a sentence. Recently, researchers have started using language models (LMs) from natural language processing (NLP) to encode the protein sequence. They have trained many protein LMs to generate embeddings for proteins, can be used as input features to deep learning models. These input features have been proven to be more valuable than the earlier approaches (substitution matrices, capturing biophysical properties) to perform tasks like protein subcellular localization and structure prediction. In this work, we explore two protein LMs models: *SeqVec*^36^ (LSTM-based) and *ProtBert*^37^ (BERT-based). The procedure of getting feature vectors using these language models is depicted in Figure 3.

The *SeqVec* is the adaption of the bi-directional language model, ELMo (Embeddings from language models)^45^ and is context-dependent. The *SeqVec* model has the same configuration as the standard ELMo architecture with some minor changes such as the reduction of tokens to 28 (20: standard amino acids, 2: rare amino acids, 3: unknown or ambiguous amino acids, 2: tokens to represent the start and end of the sequence, 1: masking). To handle the increased length of protein sequences, the number of unrolling steps has been increased. This language model consists of one character convolution (CharCNN)^46^ layer and two bi-directional LSTM layers. The CharCNN layer maps each amino acid to the fixed-length (1024) latent space. This layer does not consider information from neighbouring words. The output of this layer is the input of the first bi-LSTM layer. Each bi-LSTM unit has a dimension of 1024 (first 512 for a forward pass and the last 512 for a backward pass). The ELMo-based *SeqVec* model is trained on the UniRef50, a dataset of 33 million protein sequences. For a protein sequence with a length *L*, this pre-trained embedder generates an embedding of size (3, *L*, 1024). We get the feature vectors of this size by concatenating the outputs of 3 layers (1 CharCNN and 2 bi-LSTMs), each layer having the 1024-dimensional embedding for each word or amino acid. The per-residue embedding is generated by taking the summation of the embeddings of the 3 layers.

The second embedder that we use to get per-residue embedding (node features in a protein graph) is *ProtBert*, trained on the BFD-100 dataset. The BFD (Big Fat Database) has 2.1 billion protein sequences and more than 393 billion tokens (amino acids). The *ProtBert* LM employed the BERT model to generate descriptive features for each residue. The BERT^47^ is a deep learning model based on transformers and has been extensively used for transfer learning in NLP. For any protein sequence of length *L*, *ProtBert* LM first performs the tokenization and adds positional encoding to each token. The output of this layer is then passed through the stacked self-attention layers, which generate context-aware embeddings. Each self-attention-based encoding layer outputs an embedding of length 1024 for each residue of a protein sequence. The last hidden state of the attention stack having the dimension of (*L*, 1024) is used as a feature matrix. Since the length of the natural language sentences is much smaller when compared to that of protein sequences, some changes are made in the protein LMs, such as increasing the number of layers and unrolling steps.

## Results and analysis

This section analyzes the performance of the proposed method to predict PPI in terms of various evaluation metrics. These metrics include accuracy (*acc*), sensitivity (*Sn*), specificity (*Sp*), precision (*Pr*), *F-score*, Matthew correlation coefficient (*MCC*), the area under the precision-recall curve (*AUPRC*), and area under the receiver operating characteristic curve (*AUROC*).

### Experimental setup

We use PyTorch, an open-source deep learning framework, to build the proposed PPI models. To build GNN model variants that we explore in this work, we use PyTorch geometric. For current PPI work, the models’ performance with more than one GNN layer (e.g., 2 and 3) is either comparable to or less than that of a one-layer GNN model. So, in this work, the number of GNN layers to encode the protein graph representation is chosen to be one. The classifier module of the proposed work has two fully connected (FC) layers having *256* and *64* units, respectively. The Adam optimizer with a learning rate of *0.001* is used to minimize the MSE loss while training the model. After every FC layer in the proposed PPI model, we use a dropout layer with a dropout rate of *0.2*. The values of these parameters, such as dropout and learning rate, number of FC layers, number of units in each layer, are set following the previous works^28,33^

### Performance of GNN variants using different node features

In this work, we have represented each protein as a graph and employed different graph neural networks (GCN and GAT), which operate on these graph-structured data to get protein-level representation. The selection of input features greatly influences the performance of any model. Here, we have explored different methods to extract features for nodes of protein graphs (see Table 2). Tables 3 and 4 illustrate the performance of GNN models utilizing different node features. As visible, the residue embedding generated using LSTM-based LM yields the best results for both GCN and GAT models. For the human PPI dataset, the GAT model obtains an accuracy of *98.13%*, F-score of *98.73%*, MCC of *95.20%* and these values are slightly better than those obtained by its GCN counterpart in the respective performance metric. The GAT model for the *S. cerevisiae* dataset improves the results moderately than its GCN counterpart in terms of all evaluation metrics. From the results reported in these tables, we can observe that the performance of GNN models utilizing BERT-based LM embeddings is superior to those utilizing one-hot encoding and physicochemical properties to obtain node features. It shows the potential of language models to capture the hidden associations directly from raw sequences. The proposed model takes advantage of both graph neural networks and language models. The GNN models compute the hidden representation of a node by considering its neighboring nodes that are distant in sequence but nearer to each other in 3D space. Also, the per-residue feature extraction from sequences directly does not require background knowledge. Along with results on test data (20% of the dataset), we have also reported the average results of 5-fold cross-validation with the standard deviation of well-performing models using LSTM-based LM embeddings in Table 5 for both PPI datasets. As evident, the GAT-based classifiers performed a bit better than the GCN-based classifiers for most of the evaluation metrics.

**Table 3:** Performance of GNN variants using different node features on human test set.

GNN model	Node features	*acc*	*Sn*	*Sp*	* Pr*	* F-score*	*MCC*	*AUROC*	*AUPRC*
GCN	LSTM-based LM	97.93	98.53	**96.27**	**98.65**	98.59	94.70	**98.37**	**98.92**
	BERT-based LM	96.32	97.33	93.65	97.58	97.46	90.80	97.59	98.39
	One-hot encoding	81.30	94.24	45.46	82.72	88.10	47.47	84.25	92.97
	Physicochemical properties	77.11	95.47	26.29	78.20	85.97	31.60	75.65	88.60
GAT	LSTM-based LM	**98.13**	**98.84**	96.18	98.62	**98.73**	**95.20**	98.28	98.86
	BERT-based LM	96.59	97.52	94.15	97.77	97.64	91.48	97.35	98.21
	One-hot encoding	79.84	92.37	45.12	82.33	87.07	43.50	82.24	91.48
	Physicochemical properties	75.36	94.21	23.15	77.25	84.89	25.12	71.18	87.16

Best values are in bold.

**Table 4:** Performance of GNN variants using different node features on *S. cerevisiae* test set.

GNN model	Node features	*acc*	*Sn*	* Sp*	* Pr*	* F-score*	* MCC*	* AUROC*	* AUPRC*
GCN	LSTM-based LM	91.42	90.62	92.20	91.88	91.24	82.84	95.26	94.97
	BERT-based LM	86.68	84.33	88.95	88.07	86.16	73.40	92.01	91.43
	One-hot encoding	71.09	75.51	66.78	68.89	72.05	42.43	79.23	78.58
	Physicochemical properties	68.15	73.68	62.76	65.85	69.55	36.65	74.13	72.92
GAT	LSTM-based LM	**92.15**	**91.76**	**92.53**	**92.29**	**92.02**	**84.30**	**95.85**	**95.11**
	BERT-based LM	86.74	82.62	90.72	89.59	85.97	73.65	92.23	91.88
	One-hot encoding	69.23	64.99	73.36	70.38	67.58	38.49	75.27	71.88
	Physicochemical properties	61.15	70.14	52.40	58.94	64.05	22.88	65.40	62.64

Best values are in bold.

**Table 5:** Average results of 5-fold cross-validation of GNN variants using LSTM-based LM node features for PPI datasets.

Datasets	GNN model	* acc*	* Sn*	* Sp*	* Pr*	* F-score*	* MCC*	* AUROC*	* AUPRC*
Human	GCN	98.26 (0.39)	**99.44 **(0.52)	95.08 (1.15)	98.20 (0.47)	98.82 (0.27)	95.57 (0.76)	98.06 (0.44)	98.50 (0.42)
	GAT	**98.34 **(0.27)	99.37(0.45)	**95.54 **(0.90)	**98.37 **(0.54)	**98.87 **(0.19)	**95.76 **(0.67)	**98.38 **(0.36)	**98.96 **(0.30)
*S. cerevisiae*	GCN	94.41 (0.94)	94.19 (1.30)	**94.62 **(1.24)	**94.56 **(1.20)	94.37 (0.98)	88.82 (1.73)	97.01 (1.37)	**96.76 **(1.50)
	GAT	**94.85 **(0.76)	**95.15 **(1.45)	94.49 (1.13)	94.46 (1.15)	**94.80 **(0.87)	**89.70 **(1.52)	**97.24 **(1.20)	96.50 (1.43)

Best values are in bold.

### Effect of number of GNN layers on the performance of PPI models

One of the parameters that affects the performance of deep learning models is the number of layers^48^. In general, it is assumed that the more GNN layers there are, the more information may be gathered from the edge and node features. However, in reality, the excessive layers would cause a decrease in performance due to vanishing gradients and over-smoothing^49^. Smoothing is defined as the similarity between nodes in a graph. It is considered to be the essential nature of GNN as the nodes in the graph exchange messages with each other. When stacking multiple layers, this message interaction between them would make the representations of the nodes similar and cause over-smoothing. To demonstrate the effect of the number of GNN layers for PPI tasks, we experimented with up to 3 layers of GNN in the proposed PPI framework. The results are reported in Table 6. As evident, when we increase the number of GNN layers from one to two, the performance of PPI models (GCN/GAT-based) in terms of evaluation metrics is comparable. With three layers, the values of performance metrics are lower than those with one or two layers, except for sensitivity. Since the performance of PPI models with one and two GNN layers is comparable, we choose a model with one GNN layer.

**Figure 4:**
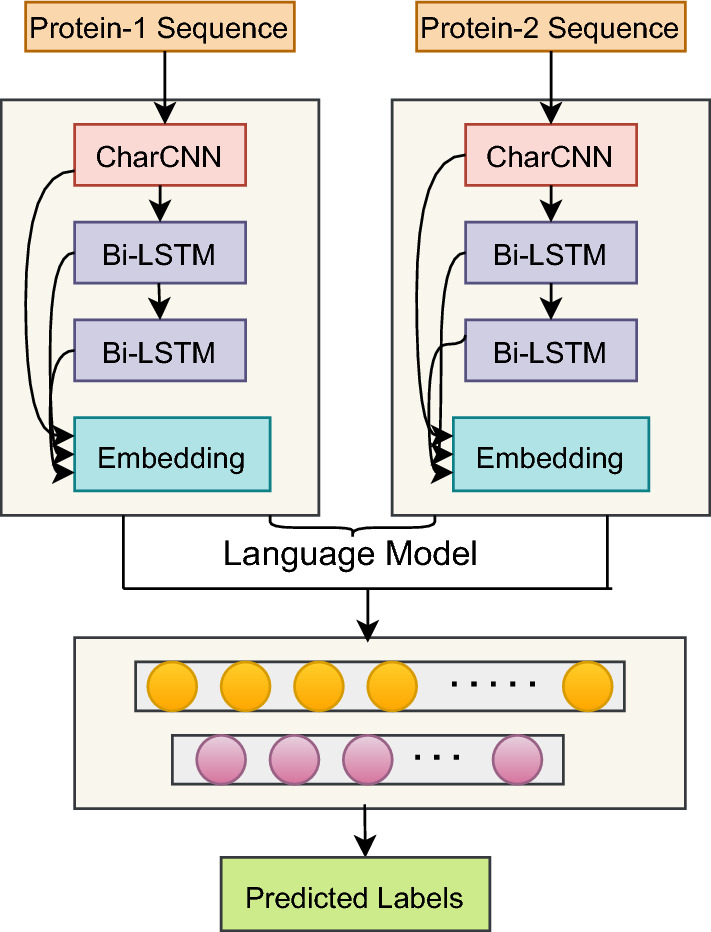
Illustration of the designed baseline.

To analyze the contribution of sequence embeddings generated using a language model (LSTM-based), we designed a baseline, depicted in Figure 4. The language model used in this work generates both *per-residue* and *per-protein* embeddings. The *per-residue* embedding is used as node features in a graphical representation of a protein. The *per-protein* embedding is generated by taking the mean across the protein sequence length. The generated embeddings for protein sequences are then fed to an MLP classifier for protein interaction prediction. The results are presented in Table 7.

**Table 6:** The results of GNN variants using LSTM-based LM node features with different number of layers for PPI datasets.

Datasets	GNN model	# layers	*acc*	*Sn*	*Sp*	* Pr*	* F-score*	* MCC*	* AUROC*	* AUPRC*
Human	GCN	1	97.93	98.53	96.27	98.65	98.59	94.70	98.37	98.92
		2	97.99	98.90	95.50	98.39	98.64	94.84	97.88	98.55
		3	97.03	98.54	93.04	97.39	97.96	92.50	96.51	97.58
	GAT	1	98.13	98.84	96.18	98.62	98.73	95.20	98.28	98.86
		2	97.73	98.13	96.61	98.77	98.45	94.21	98.24	98.92
		3	97.66	98.60	95.17	98.18	98.39	94.11	97.50	98.23
*S. cerevisiae*	GCN	1	91.42	90.62	92.20	91.88	91.24	82.84	95.26	94.97
		2	91.64	92.31	90.98	91.07	91.69	83.29	95.66	94.84
		3	90.57	92.08	89.06	89.35	90.70	81.18	94.19	93.00
	GAT	1	92.15	91.76	92.53	92.29	92.02	84.30	95.85	95.11
		2	91.76	90.39	93.09	92.72	91.54	83.53	96.30	96.06
		3	91.08	92.99	89.18	89.54	91.23	82.22	95.02	93.58

**Table 7:** The results of designed baselines using LSTM-based LM features for PPI datasets.

Datasets	* acc*	* Sn*	* Sp*	* Pr*	* F-score*	* MCC*	* AUROC*	* AUPRC*
Human	95.34	96.24	92.81	97.41	96.82	88.13	97.03	98.07
*S. cerevisiae*	89.83	90.36	89.34	88.91	89.63	79.67	94.04	93.86

### Effect of sample size on the performance of PPI models

Our method to predict PPI is based on structural information, which reduces the number of samples compared to sequence-based methods. So, to check the robustness of the current work, we train the models for different sample sizes and report the results in Table 8. In Table 8, we have reported the values of the performance metrics such as accuracy, sensitivity, specificity, and MCC, which we think are sufficient to analyze the effect of sample size on PPI models’ performance. As seen from the table, the performance of GNN models for both datasets increases as the number of samples increases. Initially, the GCN model outperforms the GAT model for both datasets, but gradually it improves the performance with more samples and yields the best results for final datasets. It is to be noted that *all samples* for the human PPI dataset are the same as reported in Table 1. But for the second dataset, following previous works of literature, we made this skewed dataset to a balanced dataset by randomly reinserting the positive samples into the original one. It gives us the *S. cerevisiae* dataset with a total number of samples of 8854, in which the ratio of positive samples to the negative samples is 1:1. For other sample sizes (Human: 4k, 8k, 12k; *S. cerevisiae*: 2k, 4k, 6k), we randomly select protein pairs from the final benchmark datasets having the fair distribution of interacting and non-interacting pairs. If we observe the values of sensitivity (*Sn*) and specificity (*Sp*) for *all samples* of the human PPI dataset, we can infer that the proposed work performs well on the skewed dataset as well. The number of positive samples is almost three times the number of negative samples for this dataset. After analyzing the patterns of the results with the sample sizes, we can say that in the future, if we have more structural data than now, we may get better results for PPI datasets.

**Table 8:** Performance analysis of GNN variants using LSTM-based LM node features for different sample sizes of PPI datasets.

Datasets	# Samples	GNN model	* acc*	* Sn*	* Sp*	*MCC*
Human	4k	GCN	93.59	94.20	92.90	87.13
		GAT	91.11	90.61	91.67	82.20
	8k	GCN	95.33	94.77	96.00	90.62
		GAT	94.60	93.96	95.36	89.16
	12k	GCN	96.72	96.83	96.60	93.42
		GAT	96.28	97.02	95.46	92.54
	all (22,217)	GCN	97.93	96.83	96.27	94.70
		GAT	98.13	98.84	96.18	95.20
*S. cerevisiae*	2k	GCN	80.00	79.49	80.49	59.97
		GAT	77.75	82.56	73.17	55.90
	4k	GCN	85.13	85.06	85.19	70.25
		GAT	85.0	85.32	84.69	70.00
	6k	GCN	89.17	89.45	88.87	78.32
		GAT	88.58	87.18	90.07	77.22
	all (8854)	GCN	91.42	90.62	92.20	82.24
		GAT	92.15	91.76	92.53	84.30

### Performance comparisons with previous work

We have considered recent works for performance comparison, including sequence-based methods^23–26,50^, multi-modal methods^28,29^, and the PPI network graph-based method^35^. Most works have reported training accuracy for human dataset (Sun’s work^23^: 97.19%, Yang’s work^35^: 99.15%). We achieve a training accuracy of more than 99.50%, which is higher than previous recent work. However, the training results are not sufficient to compare the PPI models. Therefore, we use the test set to check the predictive capability of PPI models on unseen data. Table 9 summarizes the performance comparisons between the proposed approach and previous works for the human dataset on the test set. As seen from the table, our method to predict PPI outperforms the previous methods in terms of most of the evaluation metrics, thus, indicating the proposed approach’s efficacy utilizing the GNN and LM. For the *S. cerevisiae* dataset, we have also performed the comparative study as summarized in Table 10, which also demonstrates our work’s effectiveness. Following the previous works, in Table 10 we have reported the values, which are the average of 5-fold cross-validation results. It should be noted that the current work outperforms our previous works that used a multi-modal framework in terms of the majority of the performance metrics. However, the performance improvement is not very large, but significant enough.

**Table 9:** Comparative analysis of the proposed approach with existing methods for human dataset.

Method	* acc*	* Sn*	* Sp*	*Pr*	* F-score*	* MCC*	* AUROC*	* AUPRC*
Sun’s work^23^	96.82	–	–	–	–	–	–	–
Jha’s work^29^	97.20	98.07	95.04	97.99	98.03	93.16	98.39	98.87
Yang’s work^35^	96.91	97.90	93.73	98.06	–	–	–	–
Jha’s work^28^	97.94	**98.89**	95.84	98.13	98.51	95.18	**99.18**	**99.49**
Proposed approach	**98.13**	98.84	**96.18**	**98.62**	**98.73**	**95.20**	98.28	98.86

Best values are in bold.

**Table 10:** Comparative analysis of the proposed approach with existing methods for *S. cerevisiae* dataset.

Method	* acc*	* Sn*	* Sp*	* Pr*	* F-score*	* MCC*	* AUROC*	* AUPRC*
Wong’s work^50^	93.92	91.10	–	**96.45**	–	88.60	94.00	–
Du’s work^24^	92.50	90.56	94.49	94.38	-	85.08	97.43	-
Gonzalez’s work^26^	92.59	91.40	91.59	93.65	92.51	85.20	97.40	-
Hashemifar’s work^25^	94.55	92.24	–	96.68	–	–	–	–
Jha’s work^28^	94.49	**95.79**	93.19	93.41	94.58	89.01	**98.01**	**97.27**
Proposed approach	**94.85**	95.15	**94.49**	94.46	**94.80**	**89.70**	97.24	96.50

Best values are in bold.

## Conclusion

In this work, we propose a method that combines graph neural network (GNN) and language model (LM) to predict the interaction between proteins. First, we build a molecular protein graph (amino acids/residues as nodes) from a PDB file containing structural information. Then we use LM to generate per-residue embedding from the PDB sequence, which is used as the node’s feature of the protein graph. The GNN-based model then extracts features from the protein’s graphical representation (combining structural and sequence information). Finally, we concatenate the outputs of the GNN-based model for each protein pair, and the resulting vectors are then fed to the PPI classifier. This classifier has two fully connected layers and an output layer. We have evaluated the performance of our method on two popular datasets, and the obtained results demonstrate its effectiveness. The proposed work outperforms the previous approaches, including PPI network-based graph auto-encoder model. However, PPI network-based model has the advantage over its protein structure-based counterpart in terms of the number of samples as the structural information is not available for all existing proteins. In the future, we will explore other deep learning-based approaches to learn features from protein representations (sequences and structures) such as multi-scale representation learning^51^ and intrinsic-extrinsic convolution and pooling for learning on 3D protein structures^52^.

## Data Availability

The dataset used in this study is available at http://www.csbio.sjtu.edu.cn/bioinf/LR_PPI/Data.htm.
